# Effects of a ketogenic diet on body composition and strength in trained women

**DOI:** 10.1186/s12970-020-00348-7

**Published:** 2020-04-10

**Authors:** Salvador Vargas-Molina, Jorge L. Petro, Ramón Romance, Richard B. Kreider, Brad J. Schoenfeld, Diego A. Bonilla, Javier Benítez-Porres

**Affiliations:** 1 Human Kinetics and Body Composition Laboratory, University of Málaga, Bulevar Louis Pasteur, 25, 29010 Málaga, Spain; 2 EADE-University of Wales Trinity Saint David, Málaga, Spain; 3 Research Group in Physical Activity, Sports and Health Sciences (GICAFS), Universidad de Córdoba, Montería, Colombia; 4 Research Division, DBSS INTERNATIONAL SAS, Bogotá, Colombia; 5 Exercise & Sport Nutrition Lab, Human Clinical Research Facility, Texas A&M University, College Station, TX USA; 6 Department of Health Sciences, CUNY Lehman College, New York, USA

**Keywords:** Resistance training, Female, High-fat diet, Energy intake, Fat distribution

## Abstract

**Background:**

The effect of ketogenic diets (KD) on body composition in different populations has been investigated. More recently, some have recommended that athletes adhere to ketogenic diets in order to optimize changes in body composition during training. However, there is less evidence related to trained women. We aimed to evaluate the effect of a KD on body composition and strength in trained women following an eight-week resistance training (RT) program.

**Methods:**

Twenty-one strength-trained women (27.6 ± 4.0 years; 162.1 ± 6.6 cm; 62.3 ± 7.8 kg; 23.7 ± 2.9 kg·m^− 2^) were randomly assigned to either a non-KD group (*n* = 11, NKD) or a KD group (*n* = 10, KD). Study outcomes included body composition as measured by dual-energy X-ray absorptiometry (DXA), strength levels measured using one maximum repetition (RM) in back squat and bench press (BP), and countermovement jump (CMJ) measured on a force plate.

**Results:**

A significant reduction in fat mass was observed in KD (− 1.1 ± 1.5 kg; *P* = 0.042; *d* = − 0.2) but not in NDK (0.3 ± 0.8 kg; *P* = 0.225; *d* = 0.1). No significant changes in fat-free mass were observed in KD (− 0.7 ± 1.7 kg; *P* = 0.202; *d* = − 0.1) or NKD (0.7 ± 1.1 kg; *P* = 0.074; *d* = 0.2), but absolute changes favored NKD. No significant changes in BP were observed in KD (1.5 ± 4.6 kg; *P* = 0.329; *d* = 0.2), although significant changes were noted in the squat and CMJ (5.6 ± 7.6 kg; *P* = 0.045; *d* = 0.5 and 2.2 ± 1.7 kg; *P* = 0.022; *d* = 0.6, respectively). In contrast, NKD showed significant increases in BP (4.8 ± 1.8; *P* < 0.01; *d* = 0.7), squat (15.6 ± 5.4 kg; *P* = 0.005; *d* = 1.4) and CMJ (22.0 + 4.2 cm; *P* = 0.001; *d* = 0.5).

**Conclusions:**

Findings indicate that a KD may help to decrease fat mass and maintain fat-free mass after eight 8 weeks of RT in trained-women but is suboptimal for increasing fat-free mass.

## Introduction

Dietary manipulation is an essential component for optimizing the adaptation to physical exercise; therefore, modulating the intake of certain specific nutrients, as is the case with a ketogenic diet (KD), can influence the ability to achieve physical objectives [1]. A KD is based on a marked reduction in carbohydrate consumption (i.e., ≈50 g per day or 10% of total daily caloric intake) and a corresponding increase in dietary fat (≈ 60–80% of total calories) and protein consumption (i.e., ≈ 1.2–1.5 g·kg^− 1^·d^− 1^) [2], although protein intake should be even higher during a strength-based training program. This macronutrient distribution leads to an increase in the production of ketone bodies (KB), such as acetoacetate, β-hydroxybutyrate and acetone, and consequently to the state of physiological ketosis (i.e., KB blood levels between 7 and 8 mM and blood pH of ≈7.4) [3]. The increase in KB and the subsequent physiological adaptations after following a KD not only have shown positive effects in the reduction of body mass (BM) in obese subjects [4], but also the reduction in blood concentrations of low-density lipoprotein cholesterol, triacylglycerols and glucose while an increase in high-density lipoprotein cholesterol has been reported [5]. Similarly, there is evidence of the benefits of KDs in the treatment or management of neurological diseases such as epilepsy [6–9] and certain types of cancer [10–12]. Therefore, adherence to a KD can be considered part of the therapeutic management of these pathologies.

In the context of physical performance, it can be speculated that KDs do not produce better results than carbohydrate-rich diets, although they could have limited benefits or, at least, not harmful to performance under certain scenarios [13]. Thus, a KD conceivably may be a plausible nutritional strategy in specific scenarios, such as: i) during prolonged low-intensity events predominantly reliant on fat oxidation to fuel exercise; ii) during the pre-competition carbohydrate-restriction phase prior to bodybuilding and/or physique competitions when it is advantageous to restrict carbohydrate intake; and/or iii) for individuals who prefer to low-carbohydrate diets [14]. However, recent studies indicate that adherence to KD may impair training adaptations and require additional study [15, 16].

Compared to endurance training, few studies have specifically investigated the effects of a KD on body composition and strength levels in resistance-trained subjects undertaking a resistance training (RT) program. It was previously reported that an eight-week RT program accompanied by a KD reduced fat mass (FM) and preserved fat-free mass (FFM) in trained men [17]. Similarly, other studies have shown favorable changes in body composition (↑ FFM and ↓ FM), strength and total testosterone [18]. However, these investigations have been conducted in men. Research investigating the effects of a KD on changes in body composition and cardiovascular risk markers are even less in women, and generally performed with no RT component and with obese and untrained female population [19, 20]. For instance, Jabekk et al. [21] reported positive changes in body composition (FM reduction and preservation of FFM) during a KD intervention in conjunction with RT in overweight women. Gregory et al. [22] also showed reductions in FM while FFM was maintained during a cross-training program in non-elite trained individuals (mostly women). Even though many studies on RT with different nutritional interventions (including on low-carbohydrate high-protein diets) have been performed in several women populations [23–28], there are a paucity of studies documenting the combined effects of KD and RT, particularly in resistance-trained women.

In view of the current gaps in the literature, the purpose of the present study was to evaluate the effects of a KD on body composition and strength levels in women undergoing a regimented RT program. We hypothesized that FM would be reduced and/or maintained while FFM and strength levels would be preserved in women undertaking an eight 8-week RT program in conjunction with a KD.

## Materials and methods

### Study design

This study was conducted as a randomized, parallel arm, controlled, prospective study. The independent variable was nutritional intervention. The primary outcome variables were changes in body composition.

### Participants

Twenty-one women (age = 27.6 ± 4.0 years; height = 162.1 ± 6.6 cm; body mass = 62.3 ± 7.8 kg; BMI = 23.7 ± 2.9 kg·m^− 2^) with at least 2 years of continuous RT experience volunteered to participate in this study. All participants committed to following the prescribed diet and RT protocols, monitoring during the eight-week study. Participants were informed of the possible risks of the experiment and provided written informed consent. The study was designed in accordance with the ethical guidelines of the World Medical Association Declaration of Helsinki [29]. The investigation was developed in Málaga (Spain). The first evaluation took place on April 2019 and the second measurement on June of the same year.

**Fig. 1:**
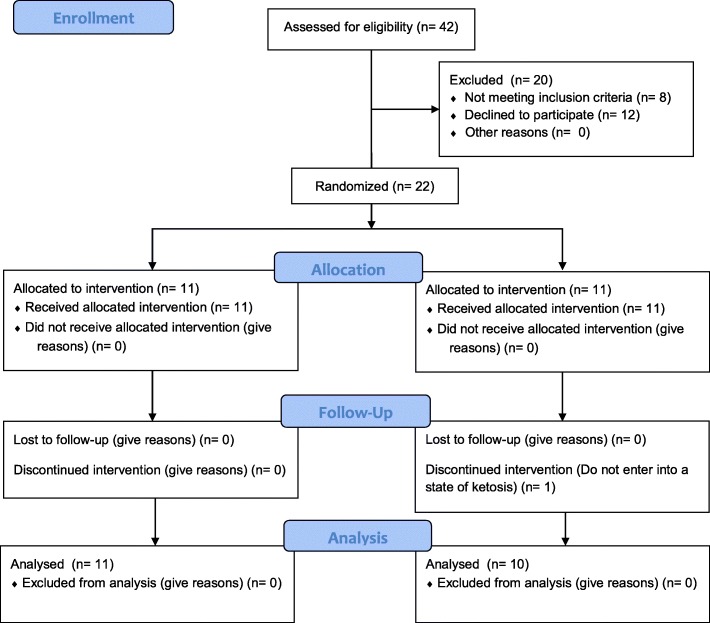
CONSORT diagram

Participants who self-reported the use of doping agents (e.g., anabolic-androgenic steroids) during the last 2 years or admitted to taking any dietary supplement during the program were excluded from participation. Women with oligomenorrhea or polycystic ovarian syndrome, as well as those not within the required age range of 18 to 35 years, were excluded. Participants were instructed to avoid performing any structured exercise during the study period other than that prescribed for the intervention. Figure 1 presents a diagram of subject enrollment as per the guidelines of the Consolidated Standards of Reporting Trials (CONSORT).

### Procedures

Participants were randomly assigned to either the KD group (*n* = 10) or the NKD group (*n* = 11), and began their training and diet at the end of the familiarization phase. The research assistants logged all training loads performed by participants for each exercise session. Strength measurements were performed 7 days after menstruation, considering this time would coincide with the follicular phase of the menstrual cycle which has been shown to be correlated to strength increases during RT [30].

#### Body composition

Body composition was measured 7 days after menstruation in both the pre- and post-intervention periods to avoid the potential for BM alterations due to water retention caused by hormonal fluctuations [31, 32]. Total body and regional body composition was estimated using dual-energy x-ray absorptiometry (DXA). Each subject was scanned by a certified technician, and the distinguished bone and soft tissue, edge detection, and regional demarcations were calculated by computer algorithms (software version APEX 3.0, Hologic QDR 4500, Bedford, MA). For each scan, participants wore sport clothes and were asked to remove all materials that could attenuate the X-ray beam. This included jewelry items and underwear containing wire. Calibration of the densitometer was checked daily against standard calibration block supplied by the manufacturer (Phantom 21,965 Lumbar Spine with anthropomorphic characteristics of 4 hydroxyapatite vertebrae included in resin. Coefficient of Variation: 0.415%).

The abdominal region was delineated by an upper horizontal border located at half of the distance between the acromion processes and external end of iliac crests, a lower border determined by the external end of iliac crests and the lateral borders extending to the edge of the abdominal soft tissue. All trunk tissue within this standardized height region was selected for analysis. To determine intertester reliability, two different observers manually selected the area for each subject.

#### Exercise protocol

The participants initially completed 3 weeks of familiarization to establish training weights for each exercise, followed by an eight-week intervention period. Cadence of repetitions was controlled by a metronome (Metronome M1, JSplash Apps). All participants performed the same exercises encompassing the major muscles of the body throughout the duration of the program. The upper limb exercises included bench presses, barbell rows, military presses, lat pulldown, incline chest presses, biceps curls and triceps pushdowns. Lower limb exercises included squats, lunges, leg presses, hip thrusts, leg extensions, lying leg curls and standing calf raises.

**Fig. 2:**
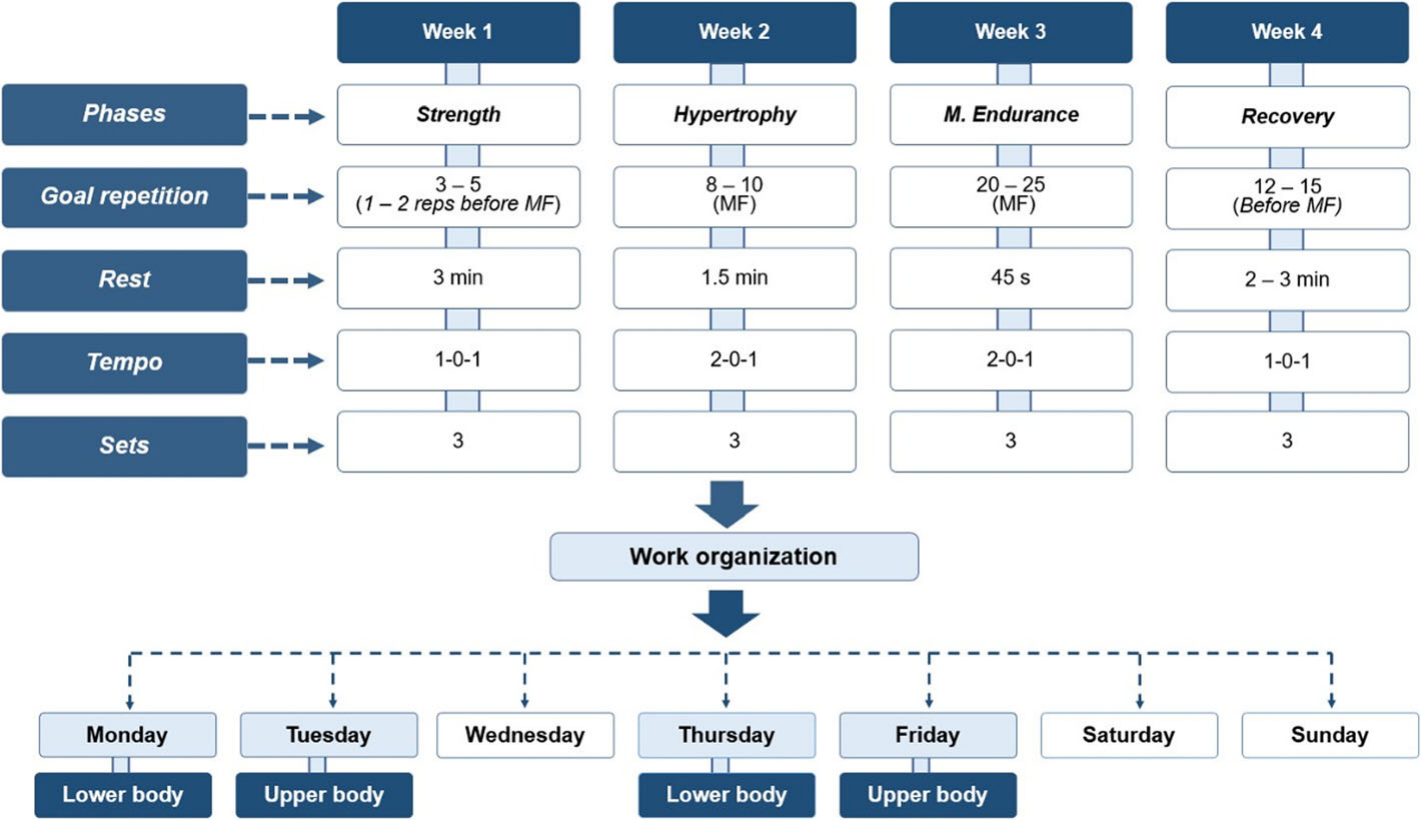
Design of strength training. 1–0-1 = a second eccentric phase, zero isometric and 1 second in the concentric and 2–0-1 = 2 seconds eccentric phase, zero isometric and 1 second in the concentric; Training phases (strength, hypertrophy and muscular endurance) and goal repetitions according to established criteria by National Strength and Conditioning Association, NSCA [33]

After familiarization, participants completed four training sessions per week (divided into 2 4-week cycles) for 8 weeks. An upper/lower body split routine was employed, with a 72-h recovery period between sessions for the same muscle complex. Both groups used a nonlinear periodized workout scheme, with the variables manipulated based on the objective of each phase as follows: strength, hypertrophy and muscular endurance. This sequence was followed by a deload whereby the volume was reduced (series x repetition x load) in the last week of each cycle (recovery phase). In total, 2 4-week cycles were completed. Figure 2 provides the specific manipulation of variables for each phase of the training cycle.

The loads were adjusted in the hypertrophy and muscular endurance phases starting with the first series of each exercise, and they were modified in subsequent series based on perceived exertion and the number of repetitions completed, to achieve concentric failure in every set and maintain the range of established repetitions. In contrast, during the week focused on strength development, participants were instructed to terminate sets 1-2 repetitions short of failure. All participants increased training loads during the first 3 weeks of each cycle as long as they were able to complete the sets without compromising technical execution.

Training sessions were monitored by RT specialists, adjusting the loads whenever necessary. The lifted loads and perceived exertion in each exercise were monitored by the strength and conditioning specialist using a paper tracking form throughout the experiment. All participants used the same exercises for the duration of the program.

#### Nutrition intervention

Diet planning was based on the participants’ FFM (g·kg-FFM^− 1^·d^− 1^) considering that they were trained subjects, did not have excess of body fat and that FFM was obtained through a valid method such as DXA. To avoid low energy availability and consequent changes in the menstrual cycle, the participants energy intake was set at ≈40–45 kcal∙kg-FFM^− 1^·d^− 1^, which is higher than that reported in previous studies (30 kcal∙kg^− 1^ lean body mass) [34, 35].

In the KD group, participants were instructed to consume 30–40 g·d^− 1^ of carbohydrates (≈20 g of dietary fiber) with a protein intake higher than the current recommendation of 1.7 g∙kg^− 1^·d^− 1^ [36]. The remaining calories were obtained in the form of dietary fats (≈31% saturated; ≈40% monounsaturated; ≈29% polyunsaturated fat). Participants were advised to eat 3-6 meals a day. To assess dietary compliance, urinary ketones concentrations were measured weekly in the early morning using over-the-counter reagent strips (*Ketostix, Bayer Vital GmbH, Leverkusen, Germany*) [37]. Alternatively, the NKD group was instructed to consume ≥1.7 g∙kg^− 1^·d^− 1^ of protein and 1 g∙kg^− 1^·d^− 1^ of fat (≈20% saturated; ≈48% monounsaturated; ≈32% polyunsaturated fat) with the remaining calories obtained from carbohydrates (≈60% starch; ≈25% simple; 15% fiber).

To monitor dietary intake, participants recorded their daily macronutrient intake via a smartphone app (MyFitnessPal, LLC, CA, USA), which has been validated as viable tool for energy and macronutrient assessment [38]. A sports nutritionist with experience in RT instructed participants on proper use of the app and managed dietary consumption over the course of the study.

#### Strength levels

For the strength tests, participants were instructed not to do any type of training during the previous 72 h, in both pre- and post-intervention. Prior to testing, participants performed a general warm-up consisting of joint mobility and 10–12 min of low-intensity aerobic exercise on exercise bike.

The Countermovement jump (CMJ) test was performed on a jump mat (Smart Jump; Fusion Sport, Coopers Plains, Australia) after instructing participants on proper jump execution. A total of 3-5 attempts were performed for familiarization prior to obtaining measurements. Participants were instructed to initiate the move by reaching 90° of knee flexion while keeping their hands at the waist and their trunk erect. Instructions emphasized that the movement should be performed without interruption from the beginning to the end of the jump. After familiarization, two jumps were recorded with a rest interval of 1 min between each; the highest value was computed.

One repetition maximum (RM) was evaluated in the squat (SQ) and bench press (BP) performed on a Smith machine (Gervasport, Madrid, Spain) exercises both at the beginning and at the end of the study. Participants reported to the laboratory having refrained from any exercise other than activities of daily living for at least 48 h before baseline testing and at least 48 h before testing at the conclusion of the study. In brief, participants performed a general warm-up before testing that consisted of light cardiovascular exercise lasting approximately 7 to 10 min. A specific warm-up set of the given exercise was performed for 12 to 15 repetitions at ~ 40% of participants’ perceived 1RM followed by two to three sets of two to three repetitions at a load corresponding to approximately 60 to 80% 1RM. Participants then performed sets of one repetition of increasing weight for 1RM determination. Three- to 5-min rest was provided between each successive attempt. Participants were required to reach parallel in the 1RM SQ; confirmation of squat depth was obtained by a research assistant positioned laterally to the subject to ensure accuracy. Successful 1RM BP was achieved if the subject displayed a five-point body contact position (head, upper back, and buttocks firmly on the bench with both feet flat on the floor) and executed full-elbow extension in the concentric phase, and the bar was required to touch the chest in the eccentric phase. 1RM SQ testing was conducted before 1RM BP with a 7-min rest period separating tests. Participants then performed as many attempts as necessary until repetition failure, using the protocol described by McGuigan [39]. Bench placement was set by marking the floor with adhesive tape, to maintain the same placement for both measurements. All testing sessions were supervised by the research team to achieve a consensus for success on each trial.

#### Statistical analysis

The statistical results are expressed as the mean ± standard deviation. The comparison between the means (pre- vs post-test) was performed with a paired t-test or the Wilcoxon test (according to data normality), and the effect size was determined using Cohen’s *d*. A repeated-measures general linear model (GLM) was used to evaluate the effect of intrasubject factors (*Time*: pre and post) and intersubject factors (i.e., the training protocol: *Group*) and the interaction between them (*Time* x *Group*). The difference in the univariate analysis of this model was established with the Greenhouse-Geisser test, also considering the partial-eta squared (η_p_^2^) value as a measure of effect size, and the Bonferroni test was used for the post hoc analysis. Additionally, a 95% confidence interval (CI) for the mean of the change from baseline (Δ = pos-test - pre-test) was used to analyze significant changes in the variables, with values above or below a 95% CI denoting significant changes. Likewise, the Mann-Whitney U test (according to data normality) was used to compare the Δ between groups. Data normality was verified with the Shapiro-Wilk test. The level of significance assumed for all tests was *P* < 0.05. The statistical procedures were performed with the statistical package SPSS version 24 (SPSS Inc., Chicago, USA), and the graphs were developed using GraphPad Prism software version 7.03 (GraphPad Software, California, USA).

## Results

In this study, 42 volunteers were identified, of whom eight did not meet initial inclusion criteria. Subsequently, 12 participants declined to participate after being informed about the type of training and the possibility of joining a group with controlled ketosis. Twenty-two participants were equally randomized to either the KD group or the NKD group. One subject was excluded from the KD group at Week 2 two for not showing positive ketosis strips. No statistically significant differences were observed in the characteristics of the participants at baseline (Table 1).

**Table 1:** Characteristics of participants at baseline

	KD (***n*** = 10)	NKD (***n*** = 11)	Total (***n*** = 21)	***p-***value
**Age (y)**	26.8 ± 3.9	28.3 ± 4.1	27.6 ± 4.0	0.41
**Height (cm)**	161.6 ± 7.4	162.6 ± 6.2	162.1 ± 6.6	0.73
**BM (kg)**	61.9 ± 9.8	62.6 ± 5.9	62.3 ± 7.8	0.51
**BMI (kg∙m**^**−2**^**)**	23.8 ± 3.6	23.7 ± 2.2	23.7 ± 2.9	0.96
**FM (kg)**	18.4 ± 6.4	18.3 ± 4.3	18.4 ± 5.3	0.98
**FFM (kg)**	42.8 ± 5.4	43.5 ± 2.8	43.2 ± 4.1	0.70
**BP (kg)**	41.5 ± 8.4	39.8 ± 7.1	40.6 ± 7.6	0.63
**Squat (kg)**	68.5 ± 11.2	64.5 ± 11.3	66.5 ± 11.1	0.44
**CMJ (cm)**	20.8 ± 2.7	22.0 ± 4.2	21.4 ± 3.5	0.45

The values are expressed as average ± standard deviation; *KD* ketogenic diet group; *NKD* non-ketogenic diet group; *BM* body mass; *BMI* body index mass; *FM* fat mass; *FFM* fat-free mass; *BP* bench press; *CMJ* countermovement jump

Considering that diet planning was based on the participants’ FFM (g·kg^− 1^ FFM·d^− 1^), the macronutrient distribution in relation to energy intake in the KD group resulted in < 10% for carbohydrates, ≈65% for fat and ≈26% of protein (see Table 2 for details), which allowed dietary adherence and led to a daily energy intake of 40.1 ± 2.7 kcal·kg-FFM^− 1^·d^− 1^ (1710.4 ± 160.0 kcal/d). On the other hand, the macronutrient distribution of the NKD group (Table 2) resulted in a daily energy intake of 45.5 ± 1.6 kcal·kg-FFM^− 1^·d^− 1^ (1979.6 ± 140.0).

**Table 2:** Energy and macronutrients intake

	KD	NKD	***p-***value
**Protein**			
g∙d^−1^	115.1 ± 17.7	97.3 ± 7.6	0.012
% kcal Total	26.8 ± 2.3	19.7 ± 1.4	< 0.05
g∙kg-FFM^−1^∙d^− 1^	2.7 ± 0.3	2.2 ± 0.1	0.002
**Carbohydrates**			
g∙d^− 1^	38.6 ± 4.5	282.1 ± 25.1	< 0.05
% kcal Total	9.1 ± 1.3	57.0 ± 1.9	< 0.05
g∙kg-FFM^− 1^∙d^− 1^	0.9 ± 0.1	6.5 ± 0.4	< 0.05
**Fat**			
g∙d^− 1^	121.7 ± 11.8	51.3 ± 4.6	< 0.05
% kcal Total	64.1 ± 2.3	23.3 ± 1.6	< 0.05
g∙kg-FFM^− 1^∙d^− 1^	2.9 ± 0.2	1.2 ± 0.1	< 0.05
**Calories**			
kcal∙kg-FFM^− 1^∙d^− 1^	40.1 ± 2.7	45.5 ± 1.6	< 0.05
kcal∙d^− 1^	1710.4 ± 160.0	1979.6 ± 140.0	0.001

The values are expressed as average ± standard deviation; *KD* ketogenic diet group; *NKD* non-ketogenic diet group; *FM* fat mass; *FFM* fat-free mass.

**Table 3:** Results before and after the intervention by groups

	Group	Before (X ± SD)	After (X ± SD)	^**a**^ (X ± SD [95% CI])	***p-***value	***d***	Time	Group	Time x Group
							*P* (η_p_^2^)		
BM (kg)	KD	61.9 + 9.8	59.7 + 10.1	−2.2 ± 1.2 [−3.0, − 1.3]*	0.005	−0.2	0.08 (0.16)	0.53 (0.02)	< 0.01 (0.52)
	NKD	62.6 + 5.9	63.4 + 6.5	0.8 ± 1.8 [−0.4, 2.0]*	0.154	0.1			
FM (kg)	KD	18.4 + 6.4	17.3 + 5.5	−1.1 ± 1.5 [−2.2, −0.1]	0.042	−0.2	0.19 (0.09)	0.74 (0.01)	0.01 (0.30)
	NKD	18.3 + 4.3	18.7 + 4.5	0.3 ± 0.8 [−0.2, 0.9]	0.225	0.1			
FFM (kg)	KD	42.8 + 5.4	42.1 + 6.1	−0.7 ± 1.7 [−1.9, 0.5] *	0.202	− 0.1	0.94 (0.00)	0.47 (0.03)	0.04 (0.21)
	NKD	43.5 + 2.8	44.2 + 3.4	0.7 ± 1.1 [−0.1, 1.4]*	0.074	0.2			
BP (kg)	KD	41.5 + 8.4	43.0 + 7.7	1.5 ± 4.6 [−1.8, 4.8]*	0.329	0.2	0.001 (0.47)	0.91 (0.00)	0.05 (0.19)
	NKD	39.8 + 7.1	44.6 + 7.4	4.8 ± 1.8 [3.6, 5.9]*	< 0.01	0.7			
Squat (kg)	KD	68.5 + 11.2	74.1 + 12.3	5.6 ± 7.6 [0.1, 11.0]*	0.045	0.5	< 0.01 (0.74)	0.84 (0.00)	< 0.01 (0.39)
	NKD	64.5 + 11.3	80.1 + 10.8	15.6 ± 5.4 [11.7, 19.4]^*^	0.005	1.4			
CMJ (cm)	KD	20.8 + 2.7	22.4 + 3.3	1.7 ± 1.9 [0.3, 3.1]	0.022	0.6	0.00 (0.56)	0.48 (0.03)	0.46 (0.03)
	NKD	22.0 + 4.2	24.2 + 4.9	2.2 ± 1.7 [1.1, 3.4]	0.001	0.5			

^a^, changes from baseline; *CI* confidence interval; *P*, significant difference between pretest vs postest; d, Cohen’s d; *KD* ketogenic diet group; *NKD* non-ketogenic diet group; *FM* fat mass; *FFM* fat-free mass; *BP* bench press; *CMJ* countermovement jump; * difference (*P* < 0.05) between KD and NKD group.

### Body composition

#### Body mass

A significant post-study decrease in BM was observed with a small effect in the KD group (*P* < 0.01, *d* = − 0.2), while the NKD group showed no significant changes (*P* = 0.154; *d* = 0.1). The change with respect to baseline (Δ) in the KD group was − 2.2 ± 1.2 kg [− 3.0, − 1.3 kg] and was considered statistically significant, while the NKD group showed a nonsignificant increase of 0.8 ± 1.8 kg [− 0.4, 2.0 kg]. Comparison of the Δ between the groups revealed a statistically significant difference (*P* < 0.01). According to the general linear model (GLM), there was no difference considering the *Time* (*P* = 0.08; η_p_^2^ = 0.16) or *Group* (*P* = 0.53; η_p_^2^ = 0.02) factors, but there was a difference in the *Time x Group* interaction (*P <* 0.01; η_p_^2^ = 0.52) (Table 3, Fig. 2a).

#### Fat mass

**Fig. 3:**
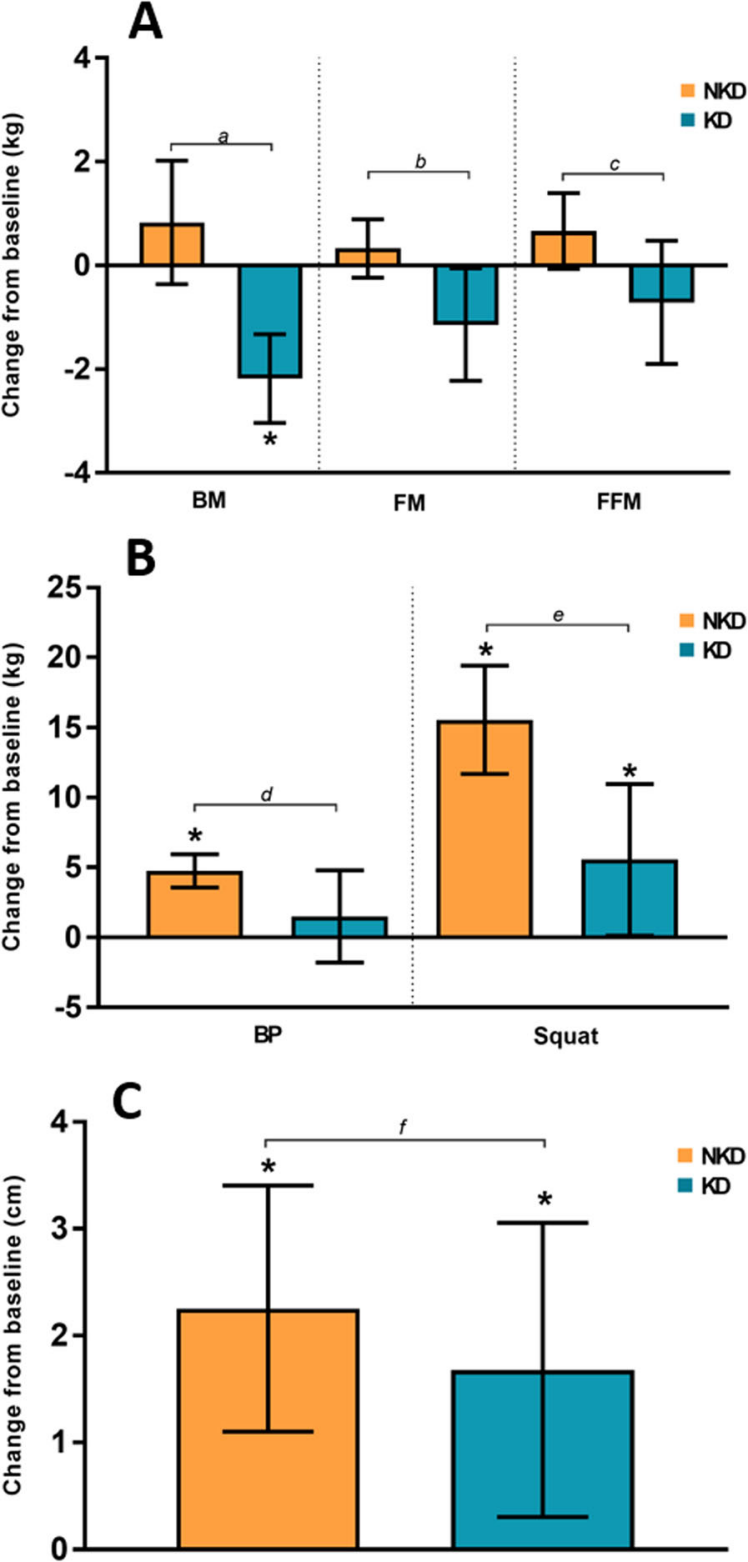
**a**. Changes from baseline in BM (body mass), FM (fat mass) and FFM (fat-free mass); **b**. Changes from baseline in BP (bench press) and Squat; **c**. Changes from baseline in CMJ. Legend: The error bar represents the confidence intervals at 95% (95% CI); * when the 95% CI completely exceeds O, it is considered a significant change. The lowercase letters represent the *P*-value of the comparison between the groups: a = < 0.01; b = 0.012; c = 0.035; d = 0.035; e = 0.003; f = 0.478

A significant post-study decrease in FM with a small effect was observed in the KD group (*P* = 0.042; *d* = − 0.2), while the NKD group showed no significant changes (*P* = 0.225; *d* = 0.1). In terms of Δ, the KD group showed a decrease of − 1.1 ± 1.5 [− 2.2, − 0.1], and the NKD group showed an increase of 0.3 ± 0.8 [− 0.2, 0.9]. The change was significant only for the KD group according to the CIs. Factor analysis revealed no changes considering *Time* (*P* = 0.19; η_p_^2^ = 0.09) or *Group* (*P* = 0.74; η_p_^2^ = 0.01), but a *Time x Group* interaction was observed (*P* = 0.01; η_p_^2^ = 0.30) (Table 3, Fig. 3a).

#### Fat-free mass

No post-study differences in FFM were observed for the KD or NKD groups (*P* = 0.202; *d* = − 0.1 and *P* = 0.074; *d* = 0.2, respectively). However, Δ showed a decrease in the KD group (− 0.7 ± 1.7 kg [− 1.9, 0.5 kg]) and an increase in the NKD group (0.7 ± 1.1 [− 0.1, 1.4 kg]), with significant differences noted between groups (*P* = 0.03). The GLM analysis revealed no differences in the *Time* or *Group* factors (*P* = 0.94; η_p_^2^ = 0.00 and *P* = 0.47; η_p_^2^ = 0.03, respectively), but *Time x Group* differences were observed (*P =* 0.04; η_p_^2^ = 0.2). (Table 3, Fig. 3a).

### Physical activity intervention

#### Bench press

No significant post-study increases in BP were found in the KD group (*P* = 0.329; *d* = 0.2), whereas the NKD group showed significant increases of a moderate effect (*P* = 0.005; *d* = 0.7). The Δ was 1.5 ± 4.6 kg [− 1.8, 4.8 kg] for the KD group and 4.8 ± 1.8 kg [3.6, 5.9 kg] for the NKD group, with significant differences shown between groups (*P* = 0.035). The GLM analysis established that there were changes considering the *Time* factor (*P* = 0.001; η_p_^2^ = 0.47) but not the *Group* factor (*P* = 0.91; η_p_^2^ = 0.00) or the *Time x Group* interaction (*P* = 0.05; η_p_^2^ = 0.19) (Table 3, Fig. 3b).

#### Squat

A significant post-study increase in Squat with a moderate effect was observed in the KD group (*P* = 0.045; *d* = 0.5), along with a significant increase with a large effect in the NKD group (*P* < 0.005; *d* = 1.4). The Δ was 5.6 ± 7.6 kg [0.1, 11.0 kg] in the KD group and 15.6 ± 5.4 kg [11.7, 19.4 kg] in the NKD group, with significant differences between groups (*P* = 0.003). The GLM analysis of factors determined that there were differences in *Time* (*P* < 0.01; η_p_^2^ = 0.74) but not in *Group* (*P* = 0.84; η_p_^2^ = 0.00). In the *Time x Group* interaction, a significant difference was observed (*P* < 0.01; η _p_^2^ = 0.39) (Table 3, Fig. 3b).

#### CMJ (countermovement jump)

A significant post-study difference was found with a moderate effect in the KD and NKD groups (*P* = 0.022; *d* = 0.6 and *P* = 0.001; *d* = 0.5, respectively). The Δ was 1.7 ± 1.9 cm [0.3, 3.1 cm] in the KD group and 2.2 ± 1.7 cm [1.1, 3.4 cm] in the NKD group, with no observed between-group (*P* = 0.478). Changes were found in the *Time* factor (*P* < 0.01; η_p_^2^ = 0.56), but not the *Group* factor or the *Time x Group* interaction (*P* = 0.48; η_p_^2^ = 0.03 and *P* = 0.46; η_p_^2^ = 0.03, respectively) (Table 3, Fig. 3c).

## Discussion

Our study aimed to evaluate the effect of a KD on body composition and strength following an 8-week RT program in strength-trained women. In light of results obtained in men under similar conditions [17], we hypothesized that a KD combined with RT would not alter FFM or strength-related parameters in strength-trained women; the results obtained partially support our hypothesis.

The use of reagent strips for the qualitative assessment of ketosis state was necessary in this study given the design and resource limitations. There were positive outcomes in every weekly test of the study (no data available since only dietary compliance was assessed, besides the dietary control during the intervention). With respect to FFM, we found no statistical pre- to post-study changes in the KD group, although an absolute decrease of 0.7 kg was observed in this outcome. Previous research using the CrossFit® training modality with mixed samples (men and women) generally lends support to our findings. Gregory et al. [40] found no statistical changes in DXA-derived measures of FFM following a 6-week RT program combined with a KD. However, the KD group was instructed to consume foods ad libitum with a maximum of 50 g of carbohydrates per day, and reported decreasing energy intake by ~ 400 cal across the study period. More recently, Kephart et al. [41] showed decreases in DXA-derived lower limb FFM and a decrease in vastus lateralis muscle thickness assessed by ultrasound when consuming a KD over a 12-week RT. The nutritional intervention of this study did not consider specific values of total calories or macronutrients used per g∙kg^− 1^·d^− 1^, impairing the ability to estimate whether participants were in a state of energy deficiency or surplus. Similarly, recent research on male military personnel have shown a reduction on BM (including FFM loss) in the KD group only, while the NKD group increased this parameter with a significant reduction in FM; notwithstanding, energy intake was not controlled in this study [42]. In contrast, Volek et al. [43] reported a statistically significant increase in FFM (1.1 kg) in moderately active male participants, some of whom reported routinely performing moderate intensity RT, following a 6-week RT program. Wilson et al. [18] also reported increases in FFM following a KD combined with intensive RT in trained men. It is important to note that the evaluation of FFM by DXA includes the intracellular water, which is stored in concert with muscle glycogen in a ~ 3:1 ratio [44]. Thus, implementing a carbohydrate refeed to the KD in the post-evaluation conceivably would positively influence the final FFM results, as demonstrated by Wilson et al. [18].

Physiological differences between sexes can be problematic when attempting to extrapolate data from men to women and vice-versa. The issues are highlighted in a recent study that investigated the effects of a four-week KD on the utilization of fats and carbohydrates during an incremental cycling test in CrossFit®-trained men and men [45]. Results showed that men increased their utilization of fats to a greater extent than women during submaximal intensity exercise, suggesting some adaptations from a KD are sex-specific.

Despite our attempts to create an energy surplus, participants in KD showed signs of satiety from the fifth/sixth week of the protocol. As shown in Table 2, reported energy consumption for participants in KD was below prescribed amounts, revealing the difficulty of eating in a caloric surplus when carbohydrate intake is severely restricted; notwithstanding, it is important to note that both groups met the daily energy intake of 40–45 kcal∙kg-FFM^− 1^. Given that the KD group showed a mean reduction in both FM and FFM, it therefore can be concluded that these participants were in an energy deficit despite claiming adherence to the nutritional prescription. It has been previously established that KDs generate a decrease in appetite [46, 47], which can be conducive to reducing FM. However, if a KD-induced state of satiety prevents individuals from consuming the prescribed caloric intake, it could be suboptimal for increasing FFM [48, 49]. Furthermore, it should be noted the possibility that KD induces gluconeogenesis, which might reduce FFM by breaking down the endogenous proteins in a higher rate.

On the other hand, this study showed significant changes for lower-limb strength (1RM squat) in both KD and NKD groups; however, only the NKD group had significant changes on the upper-body strength (1RM BP). These results partially support the work of Wilson et al. [18] where, considering the difference in sex, the researchers found significant changes when compared to baseline values of both 1RM squat and BP in a KD and NKD group (with no differences between groups). Kephart et al. also found no negative results on the 1RM back squat when compared to a NKD group. Recently, it has been reported similar changes on anaerobic parameters related to lower and upper-limb strength and power between a KD and NKD groups [42]. In fact, our study also showed similar increases on power (CMJ) after the dietary interventions in both groups from baseline. At no time point were there any differences between conditions for peak power.

### Limitations

This study has several flaws and limitations. First, the use of urine ketone strips may be less sensitive than blood-sample methods, which represents a potential methodological limitation. Also, participants of the KD group displayed signs of reduced appetite that impaired their ability to consume the same number of daily calories in comparison to the NKD group; thus, we cannot confidently extrapolate findings to how an energy surplus affects body composition and strength adaptations in KD combined with regimented RT. However, it should be noted that at both groups met the ⁓40–45 kcal∙kg-FFM^− 1^·d^− 1^. Moreover, *pre-study* measures of body composition were performed when glycogen levels were high, whereas *post-study* measures were conceivably performed in a glycogen-depleted state for KD. Given that each gram of glycogen stores at least ~3 grams of water, this could influence data on FFM as body water is incorporated into DXA-derived measures. Finally, the relatively small sample size (*n* = 21) and short intervention time (8 weeks) reduce the ability to draw strong conclusions on studied outcomes.

## Conclusions

Our results suggest that an 8-week is a viable option for decreasing FM and maintaining FFM when combined with regimented RT KD in strength-trained women; however, it is suboptimal for increasing FFM.

## Data Availability

The datasets used and analyzed during the current study are available from the corresponding author on reasonable request.

## Abbreviations

BMI: Body mass index
BP: Bench press
BM: Body mass
CI: Confidence interval
CG: Control group
CMJ: Countermovement jump
DXA: Dual-energy x-ray absorptiometry
ES: Effect size
FM: Fat mass
FFM: Fat-free mass
GLM: General lineal model
KB: Ketogenic bodies
KD: Ketogenic diet
NKD: Non-ketogenic diet
ηp2: Partial eta squared effect size
RM: One maximum repetition
RT: Resistance training
SQ: Squat
